# Hydrogenations and electric field induced magnetic behaviors in armchair silicene nanoribbons

**DOI:** 10.1038/srep23677

**Published:** 2016-03-30

**Authors:** Dan Zhang, Mengqiu Long, Fang Xie, Jun Ouyang, Hui Xu, Yongli Gao

**Affiliations:** 1 Institute of Super-microstructure and Ultrafast Process in Advanced Materials, School of Physics and Electronics, Central South University, Changsha 410083, China; 2 Physical Science and Technology College of Yichun University, Yichun 336000, China; 3 Department of Physics and Astronomy, University of Rochester, Rochester, NY 14627, United States

## Abstract

Using the first-principles calculations, we investigate the geometric, electronic and magnetic properties of armchair silicene nanoribbons with different edge hydrogenations. Our results show that the interesting magnetic behaviors such as the bipolar magnetic semiconductor can be found. Moreover, the addition of the transverse electric field can modulate the bipolar magnetic semiconductor to half-metal or spin-splitting metal. And the spin-up electrons are localized at one edge, the spin-down holes localized at the opposite edge under the external electric field. These results may present a new avenue for band engineering of silicene nanoribbons and benefit the design of silicon-based nano-spin-devices in nanoelectronics.

In the past decade, the successful preparation of one-atom-thick two-dimensional honeycomb graphene^1^ opens an approach for the nano-sized electronic devices since it possesses many excellent and unusual characteristics, such as massless Dirac fermions^2^, high carrier mobility^3^, magnetic zigzag edges^4^, long phase coherence lengths^5^, quantum Hall effect^6^, unique transport properties^7^ and so on^8^,^9^,^10^. However, graphene is a semi-metal with zero band gap, which limits its utilization in electronic devices. Furthermore, it’s incompatibility with current silicon-based electronic technology also places obstructions on the road to the brave-new-world of graphene-based devices. Hence, great efforts have been devoted to either opening an appropriate band gap in graphene^11^ or searching for other two-dimensional materials with favorable carrier mobility and opportune band gap^12^. Silicene, a material isostructural to graphene but with atomic bonds that are buckled rather than flat, has recently been synthesized and attracts enormous attentions of researchers^13^,^14^,^15^,^16^,^17^. It features a Dirac-like electron dispersion at the K points of the Brillouin zone and exhibits exciting properties beyond those present in graphene. For example, the quantum spin Hall effect induced by spin-orbit interaction^18^, a small band gap opening in experimental device^19^.

Recently, the silicene nanoribbons (SiNRs) have been synthesized on Ag(100) and Ag(110) surfaces^20^,^21^, respectively. Similar to graphene nanoribbons (GNRs), SiNRs can also be mainly classified into two types: zigzag silicene nanoribbons (ZSiNRs) and armchair silicene nanoribbons (ASiNRs). As to the ZSiNRs, they have stable antiferromagnetic (AFM) configuration, and the band structures can be tuned to half-metallic property under an external transverse electric field^22^, which have attracted numerous attentions. And a lot of other methods have been used to tune the electronic properties^23^, such as doping^24^ and edge decoration^25^. For ASiNRs, the intrinsic electronic structures show semiconductor with no magnetism and the band gap oscillates with a period of three when the ribbon width increases^26^. Presently, a few researches pointed out that, the magnetism and spin-splitting band in ASiNRs can be realized by doping. For example, Bezanilla *et al*.^27^ found nitrogen (N) atoms doping can make the ASiNRs exhibit a net magnetic moment. Zhang *et al*.^28^ studied the electronic and magnetic properties of both ASiNRs and ZSiNRs doped with a single phosphorus (P) atom, get the conclusion that the ASiNRs with P doping at edge site change into ferromagnetic (FM) semiconductor.

Moreover, the edge hydrogen saturation is also an effective way to modulate the magnetism and electronic structures of nanoribbons^25^,^29^,^30^. So, in this letter, we modulate the magnetism and electronic properties of ASiNRs by different edge hydrogenations and the bipolar magnetic semiconductor (BMS) have been found. In addition, it can also be changed to half-metal or spin-splitting metal under different external electric field. More interestingly, the charge carriers are not only spin polarized in energy space but also spatially separated at different edges under the external electric field.

## Results and Discussion

**Figure 1:**
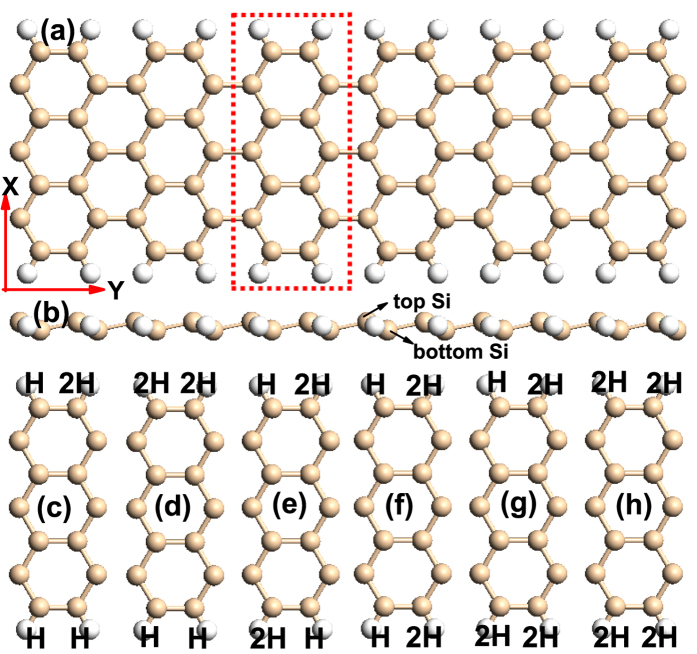
(**a**) The top view of 7-ASiNRs saturated with monohydrogen. The structure in red dashed rectangle is 7-ASiNRs unit cell (i.e. M1_(7A)_). (**b**) The side view of 7-ASiNRs. (**c–h**) The unit cells of different edge saturated 7-ASiNRs, labeled as M2_(7A)_–M7_(7A)_, respectively. The pink and white balls represent Si and H atoms, respectively.

ASiNRs can be classified by the number of silicon (Si) atoms along the ribbon width, noted as *n*-ASiNRs, as shown in Fig. 1, the width of *n* = 7 has been chosen in here (1.41 nm). The bare edged SiNRs is extremely reactive due to the dangling bonds of edged Si atoms and will be undergo reconstruction to lower the total energy^31^ and hydrogen (H) is the most common element to saturate the edged Si atom. What’s more, edge functionalization can also improve the performance of SiNRs and thus extend their applications in electronics. There are two kinds of saturated manners: monohydrogenation (*i.e. sp*^2^ edge) and dihydrogenation (*i.e. sp*^3^ edge). So the following we have a study about the electronic properties of different saturated manners of the ASiNRs unit cell. Fig. 1(a) shows the structure of single H saturated 7-ASiNRs, the red dashed rectangle represents the unit cell (marked as M1_(7A)_). The pink and white balls denote Si and H atoms, respectively. The side view of 7-ASiNRs is present in Fig. 1(b), which can be found that the Si atoms are in different planes, corresponding to two different sublattices. The Si atoms at the bottom plane are labeled as A kind sublattice and those at the top plane are labeled as B kind sublattice, as shown in Fig. 1(b). According to the percentage of *sp*^3^-like edged bonds and the kinds of saturated sublattice, there are other six models. The relaxed structures are shown in Fig. 1(c–h), which named as M2_(7A)_, M3_(7A)_, M4_(7A)_, M5_(7A)_, M6_(7A)_, and M7_(7A)_ for short, respectively.

In order to have an understanding about the stabilities of the ASiNRs with different edge hydrogenations, we calculate their formation energies, which are based on the following formula^25^,


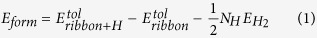


here the 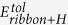, 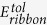, *N*_*H*_ and 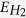 are represent the total energy of the different edge hydrogenated 7-ASiNRs, the total energy of the bare edged 7-ASiNRs, the number of H atoms, and the energy of the isolated H_2_ molecule, respectively.

The formation energies of different systems are shown in Table 1. It is shown that all the formation energies are negative, which implies that the formation of 7-ASiNRs with edge hydrogenation is an exothermic reaction from the bare edged 7-ASiNRs and H_2_, thus the edge hydrogenation can effectively enhance the stability of ASiNRs. Comparing different H saturated 7-ASiNRs, it is clear that the stability is enhanced with the number of edge dihydrogenated Si atoms (*sp*^3^ bonds) increasing. For the edge monohydrogenated structure, the bond length between the edged Si atoms is 2.23 Å. When one of the adjacent edged Si atoms is saturated by a single H atom and the other is saturated by double H atoms, the bond length increases to 2.33 Å. Once the edged Si atoms are dihydrogenated, the bond length becomes 2.35 Å.

Furthermore, we consider three different spin-polarized manners: nonmagnetic (NM), ferromagnetic coupling in each edge and between two edges (FM), and ferromagnetic ordering along each edge and antiparallel spin orientation between the two edges (AFM) for M1_(7A)_–M7_(7A)_. After having a comparison about the energy difference between three spin-polarized states, we get the ground states of each structure, as listed in Table 1. The results show M1_(7A)_, M3_(7A)_, M4_(7A)_, and M7_(7A)_ are NM, while M2_(7A)_, M5_(7A)_ and M6_(7A)_ systems are FM with the magnetic moment of 1.00 *μ*_*B*_, 2.00 *μ*_*B*_ and 1.00 *μ*_*B*_, respectively. Addition, the effect of the nanoribbon width have also been considered, the ground states and corresponding magnetic moments for different edge hydrogenated 6-ASiNR and 8-ASiNR are calculated, and the similar conclusions can also be gotten.

**Figure 2:**
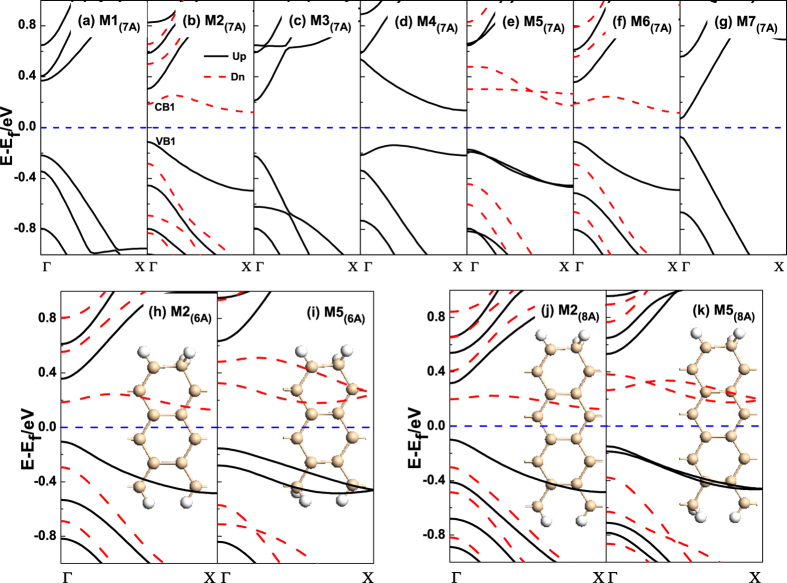
(**a–g**) The band structures of M1_(7A)_–M7_(7A)_ systems in their ground states, (**h**–**k**) The band structures of M2_(6A)_, M5_(6A)_, M2_(8A)_ and M5_(8A)_ with FM state, respectively. The blue dashed line denotes the FL.

To explore their electronic properties, we then present the band structures of M1_(7A)_–M7_(7A)_ under their ground states, as shown in Fig. 2. For M1_(7A)_, the four edged Si atoms are saturated by one H atom, we can find the band structure presents semiconductive properties with an energy gap of 0.59 eV, which agrees well with the previous study^32^. When one of the edged Si atoms is saturated by two H atoms, as to M2_(7A)_, the band structure is changed a lot. The spin-up bands (black solid line) and spin-down bands (red dashed line) are completely separated from each other, meanwhile, around the Fermi level (FL), we can find the highest valence band (VB1) and the lowest conduction band (CB1) are spin-up and spin-down states, respectively, which indicates M2_(7A)_ is a BMS^33^. When two of the edged Si atoms are separately saturated by two H atoms, and the other two are saturated by one H atom, there are three kinds of manners, namely M3_(7A)_, M4_(7A)_ and M5_(7A)_. The corresponding band structures are presented in Fig. 2(c–e), respectively. One can see that M3_(7A)_ and M4_(7A)_ are non-magnetic systems with semiconductive properties. Differently, M3_(7A)_ is a semiconductor with a direct band gap of 0.43 eV, while M4_(7A)_ is a semiconductor with an indirect band gap of 0.27 eV. When it comes to M5_(7A)_, one can find it is a BMS, and there are two spin-up (spin-down) intersected subbands below (above) the FL. When the three edged Si atoms in a unit cell are dihydrogenated, and only one Si atom is monohydrogenated, we can see the band structure of M6_(7A)_ is similar to that of M2_(7A)_. For the fully edge dihydrogenated 7-ASiNRs (M7_(7A)_), the magnetism is disappeared, meanwhile the system presents semiconductive property with a direct band gap of 0.14 eV. From M1_(7A)_–M7_(7A)_, we can conclude that the band gap is decreased with the increase of *sp*^3^-like edged bonds in 7-ASiNRs. And this would supply an effective way for us to modulate the energy gap of ASiNRs, which is similar to that in graphene nanoribbons^34^.

As reported by Cahangirov *et al*.^26^, the band gap of pristine ASiNR oscillates with a period of three when the ribbon width increases. So we also have a calculation about different edge hydrogenated 6-ASiNR and 8-ASiNR. Following the edge hydrogenations of M2_(7A)_ and M5_(7A)_, we also construct four new models for 6-ASiNRs and 8-ASiNR, named as M2_(6A)_, M5_(6A)_, M2_(8A)_ and M5_(8A)_, whose band structures are shown in Fig. 2(h–k), respectively. For M2_(6A)_, as presented in Fig. 2(h), it is clear that the CB1 and VB1 with opposite spin appear around the FL, and M2_(6A)_ is a BMS. Then for M5_(6A)_, there are two couple of opposite spin subbands appear above and below the FL, respectively. M2_(8A)_ show similar band structure with that in 6-ASiNRs and 7-ASiNRs, and the band of M5_(8A)_ is also similar with that in 6-ASiNRs and 7-ASiNRs. Thus, we can see that the BMS in ASiNRs is independent on the ribbon width.

Interestingly, we can found the M2_(7A)_, M5_(7A)_ and M6_(7A)_ are BMS and independent on the ribbon width, which indicates that the ASiNRs would have potential utilization in spin devices. According to Lieb’s theorem^35^, if a short-range inter-electron repulsion and the Hubbard U is considered, the magnetic moment and the number of spin split subbands around the FL per unit cell are determined by the number of sublattice difference: |*N*_*A*_ − N_*B*_| (*N*_*A*(*B*)_ is the number of A(B) kind sublattice)^36^. And one can obtain the magnetism once |*N*_*A*_ − *N*_*B*_| ≠ 0^37^,^38^. For M1_(7A)_, each edged Si atoms in the unit cell are saturated by one H atom, both the A and B kinds of sublattice are 7, and |*N*_*A*_ − *N*_*B*_| = 0, so M1_(7A)_ is a non-magnetic semiconductor. When one of the edged Si atom are saturated by two H atoms (as M2_(7A)_), |*N*_*A*_ − *N*_*B*_| = 1, thus the magnetic moment with 1.00 *μ*_*B*_ appears. Similarly, for M5_(7A)_, owing to both Si atoms with dihydrogenation are belong to the same sublattice, we can get |*N*_*A*_ − *N*_*B*_| = 2, so two couple of spin-resolved subbands appear near the FL, the system is magnetized with a magnetic moment of 2.00 *μ*_*B*_.

**Figure 3:**
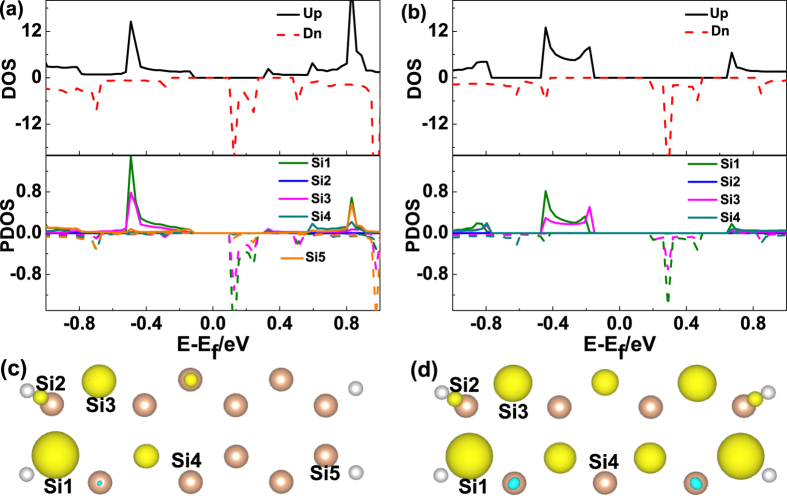
(**a**,**b**) The DOS and PDOS for M2_(7A)_, M5_(7A)_. (**c**,**d**) The spin-density distributions for M2_(7A)_, M5_(7A)_. The yellow (blue) regions correspond to the spin-up densities are bigger (smaller) than the spin-down densities. And the isovalue is 0.03Å^−3^.

To have an understanding about the origins of the spin-up (spin-down) subbands below (above) the FL (Fig. 2(b,e)), the density of state (DOS) and projected density of state (PDOS) of M2_(7A)_ and M5_(7A)_ have been plotted in Fig. 3(a,b), respectively. For M2_(7A)_, we can see the spin-down DOS appears a peak above the FL, while the spin-up DOS is non-localized below the FL, which corresponding to the two subbands near the FL (shown in Fig. 2(b)), respectively. From PDOS, one can see the DOS around the FL are mainly come from Si1 and Si3, which are next to the Si atom saturated by two H atoms, as plotted in Fig. 3(c). When it comes to M5_(7A)_, there is a peak of spin-up (down) DOS below (above) the FL, corresponding to the four subbands near the FL (shown in Fig. 2(e)). Due to the two edges are the same, so we only take one of the parts of M5_(7A)_ as a representative. One can see the DOS around the FL are mainly from Si1 and Si3. So we can get that, in M2_(7A)_ and M5_(7A)_, the subbands around the FL are mainly contributed by the Si atoms adjacent to dihydrogenated Si atoms. Fig. 3(c,d) show the spin-density distributions of M2_(7A)_ and M5_(7A)_, from which we can see the spin-density are mainly concentrated on the Si atoms adjacent to the dihydrogenated ones, and the spin-up density are much greater than that of the spin-down.

**Figure 4:**
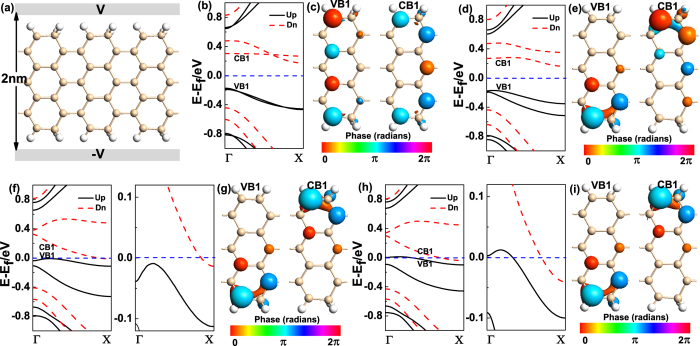
(**a**) The schematic of M5_(7A)_ with two metallic regions, which is built to apply an external transverse electric field across the nanoribbon. (**b**,**d**,**f**,**h**) The band structures of M5_(7A)_ with E_ext_ = 0.00, 0.50, 2.00, 2.25 V/nm, respectively. (**c,e,g,i**) The Bloch states of VB1 and CB1 for M5_(7A)_ at X point with E_ext_ = 0.00, 0.50, 2.00, 2.25 V/nm, respectively. The blue dashed line denotes the FL.

Furthermore, we also consider the effects of the external transverse electric field to the magnetism and electronic properties of M5_(7A)_. The external transverse electric field is applied through two metallic regions on both edges of the ribbon and tuned by the electrostatic potential of the metallic regions, the orientation of electric field is along x axis, as shown in Fig. 4(a). Our calculations show the addition of external transverse electric field can influence the magnetic moment of M5_(7A)_ observably. When the external transverse electric field is smaller than 1.00 V/nm, the magnetic moment of M5_(7A)_ keeps 2.00 μ_B_. When the external transverse electric field is in the region of [1.00, 2.50] V/nm, the magnetic moment decreases rapidly from 2.00 μ_B_ to 1.17 μ_B_ with the electric field increases. For the band structures of M5_(7A)_ under external transverse electric field, we can find the two intersected spin-up (spin-down) subbands below (above) the FL are split away from each other, and the spin band gap between VB1 and CB1 decreases with the electric field increases, as presented in Fig. 4(d,f,h). When the applied electric field increases to 2.00 V/nm (Fig. 4(f)), the CB1 goes across the FL, while the VB1 still below the FL, so the M5_(7A)_ presents half-metallic property. And the M5_(7A)_ then converts to spin-split metal when the electric field is 2.25 V/nm, as plotted in Fig. 4(h), both the VB1 and CB1 go across the FL.

We also give the Bloch states of VB1 and CB1 for M5_(7A)_ at X point with E_ext_ = 0.00, 0.50, 2.00, 2.25 V/nm, as shown in Fig. 4(c,e,g,i), respectively. When the E_ext_ = 0.00 V/nm, we can find the Bloch states of VB1 and CB1 are mainly distributed on the A kind of sublattices. And the states of VB1 are mainly localized in the left part, while that of CB1 are concentrated on the right part of M5_(7A)_. Nevertheless, this situation is broken by the applied electric field, as shown in Fig. 4(e,g,i), we can find the Bloch states are separated spatially, and independent of the strength of the electric field. The Bloch states of VB1 are mainly in one of the edges; meanwhile, those of CB1 are distributed on the other edge of M5_(7A)_. As a result, the charge carriers are not only spin polarized in energy space, but also spatially separated at different edges under the external electric field, which would supply a new method to separate electrons and holes with different spins.

## Conclusions

In conclusion, the stability, magnetic and electronic properties of ASiNRs with different H terminations have been studied by using the first-principles calculations. The results show that the stability of ASiNRs increases with the increase of the *sp*^3^-like edged bonds. Meanwhile, the ASiNRs with different edge hydrogenated manners present different electronic properties, and the BMS can be found. What’s more, the BMS can be changed to half-metal and then to spin-splitting metal as the addition external transverse electric field increase. More interesting, the states of the spin-up electrons and those of the spin-down holes are localized at the opposite edges under the external electric field. These results may be helpful in the fields of band gap tuning engineering and the designing of ASiNRs-based spin devices with control over the spin in spintronics.

## Methods

The geometry optimization and electronic properties calculation are performed within the density functional theory by using the Atomistix ToolKit (ATK)^39^,^40^ package. The wave function is expanded with the double-ζ plus polarized basis set. The Generalized gradient approximation with the Perdew-Burke-Ernzerh exchange correlation functional is adopted to describe the exchange correlation interaction. All self-consistent calculations are performed with the plane-wave cutoff energy of 200 Ry on a 1 × 21 × 1 Monkhorst-Pack k-point mesh. A vacuum layer of 20 Å is added along x and z axis to eliminate the interaction between the adjacent slabs. The geometrical structures are fully optimized until the force tolerance on each atom is smaller than 0.01 eV/Å before other quantities are calculated.

## Additional Information

**How to cite this article**: Zhang, D. *et al*. Hydrogenations and electric field induced magnetic behaviors in armchair silicene nanoribbons. *Sci. Rep*. **6**, 23677; doi: 10.1038/srep23677 (2016).

## Figures and Tables

**Table 1:** The formation energy, ground state, and corresponding magnetic moment of M1_(7A)_–M7_(7A)_.

	M1_(7A)_	M2_(7A)_	M3_(7A)_	M4_(7A)_	M5_(7A)_	M6_(7A)_	M7_(7A)_
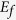 *(eV)*	−3.68	−3.93	−5.24	−4.36	−4.15	−5.47	−6.75
Ground state	NM	FM	NM	NM	FM	FM	NM
*M (μ*_*B*_)	0.00	1.00	0.00	0.00	2.00	1.00	0.00
